# The serotonergic system in Parkinson’s patients with dyskinesia: evidence from imaging studies

**DOI:** 10.1007/s00702-017-1823-7

**Published:** 2017-12-20

**Authors:** Gennaro Pagano, Flavia Niccolini, Marios Politis

**Affiliations:** Neurodegeneration Imaging Group, Maurice Wohl Clinical Neuroscience Institute, Institute of Psychiatry, Psychology and Neuroscience (IoPPN), King’s College London, 125 Coldharbour Lane, Camberwell, London, SE5 9NU UK

**Keywords:** Serotonergic system, Parkinson’s diseases, Levodopa-induced dyskinesias, Graft-induced dyskinesias, Molecular imaging, Positron emission tomography

## Abstract

The purpose of review is to review the current status of positron emission tomography (PET) molecular imaging of serotonergic system in Parkinson’s patients who experience levodopa-induced (LIDs) and graft-induced dyskinesias (GIDs). PET imaging studies have shown that Parkinson’s disease is characterized by progressive loss of dopaminergic and serotonergic neurons. Parkinson’s patients who experienced LIDs and GIDs have an aberrant spreading of serotonergic terminals, which lead to an increased serotonergic/dopaminergic terminals ratio within the putamen. Serotonergic terminals convert exogenous levodopa into dopamine in a non-physiological manner and release an abnormal amount of dopamine without an auto-regulatory feedback. This results in higher swings in synaptic levels of dopamine, which leads to the development of LIDs and GIDs. The modulation of serotonergic terminals with 5-HT_1A_ and 5-HT_1B_ receptors agonists partially reduced these motor complications. In vivo PET studies confirmed that abnormal spreading of serotonergic terminals within the putamen has a pivotal role in the development of LIDs and GIDs. However, glutamatergic, adenosinergic, opioid systems, and phosphodiesterases 10A may also play a role in the development of these motor complications. An integrative multimodal imaging approach combining PET and MRI imaging techniques is needed to fully understand the mechanisms underlying the development of LIDs and GIDs.

## Introduction

According to Braak’s staging of Parkinson’s, the pathological processes begin early in the serotonergic raphe nuclei, prior to the onset of motor symptoms, and precede the damage of substantia nigra (Braak et al. 2003). Post-mortem studies (Buddhala et al. 2015; Kish et al. 2008) have shown that the serotonergic system is affected in Parkinson’s disease, with loss of synaptic terminals in serotonin-containing neurons (Paulus and Jellinger 1991).

Positron emission tomography (PET) is a molecular imaging technique that allows the quantification of biological targets and pathological processes in vivo in the brain (Pagano et al. 2016; Politis and Piccini 2012). PET radiotracers are able to identify alterations at molecular level and have provided invaluable insight into the mechanisms underlying Parkinson’s disease. As such, PET imaging has been validated in vivo to evaluate the serotonin system in patients with Parkinson’s disease (Politis and Loane 2011; Politis and Niccolini 2015). [^11^C]-3-Amino-4-(2-dimethylaminomethylphenylsulfaryl)-benzonitrile ([^11^C]DASB) is a second-generation PET ligand with high selectivity for the serotonin transporter (SERT) (Houle et al. 2000), which has been validated to in vivo quantify serotonergic terminals.

We recently performed a meta-analysis of the in vivo evidence of serotonergic terminals integrity in Parkinson’s patients using [^11^C]DASB PET imaging (Pagano et al. 2017a, b). Serotonergic system has been investigated in 234 Parkinson’s patients across 20 PET studies. Parkinson’s patients have a reduction of serotonergic terminals in raphe nuclei, thalamus, hypothalamus, ventral striatum, caudate, and putamen, which correlates with the duration of the disease. This suggests that the progressive loss of synaptic terminals in Parkinson’s disease affects the serotonergic system and is not only confined to the dopaminergic terminals. However, Parkinson’s patients who experienced levodopa-induced dyskinesias (LIDs) showed preserved serotonergic terminals compared to Parkinson’s patients with stable response to levodopa. These findings indicate that although serotonergic system progressively degenerates in Parkinson’s disease, those who develop LIDs have an aberrant spreading of serotonergic terminals or a faster degeneration of dopaminergic than serotonergic terminals.

The mechanisms underlying LIDs are complex and still unclear. There is wide consensus that this motor complication is determined by both pre- and post-synaptic mechanisms, which converges to generate pre-synaptically large dopamine swings in the putamen concomitant with the peaks of plasma levodopa levels, while post-synaptic changes engender abnormal functional responses in dopaminoceptive neurons, associated with augmented signaling cascades (Cenci 2014). While this general picture is well accepted, the relative contribution of different factors remains a matter of debate. A particularly animated debate has been growing around putative players on the pre-synaptic side of the cascade (Cenci 2014). Dyskinesias have been also reported in Parkinson’s disease patients who underwent to intrastriatal transplantation of foetal ventral mesencephalic tissue (Politis 2010; Politis et al. 2011; Politis et al. 2010). These involuntary movements have been defined as graft-induced dyskinesias (GIDs) and occur in the absence of dopaminergic medication. GIDs might be the consequence of abnormal release of dopamine by graft-derived dopaminergic overgrowth or by serotonergic terminals. The serotonergic component of GIDs has been also hypothesized, because serotonergic neurons have been found at post-mortem in the grafted tissue of Parkinson’s Disease patients (Mendez et al. 2008), but whether these serotonergic neurons contribute to GIDs in humans is still under debate.

PET imaging has been employed for the in vivo evaluation of pre- and post-synaptic mechanisms involved in the development of LIDs in Parkinson’s disease (Niccolini et al. 2015). We have extensively investigated the pre-synaptic role of serotonergic terminals in the development of LIDs (Politis et al. 2014; Roussakis et al. 2016; Smith et al. 2015) and in Parkinson’s disease (Politis 2010; Politis et al. 2010, 2011).

This review discusses the current status of PET molecular imaging of serotonergic system in Parkinson’s patients who experiences LIDs and GIDs, and its relation with the underlying mechanisms of Parkinson’s disease.

### The role of serotonergic system in development of LIDs in Parkinson’s disease: the theory and the experiments in preclinical models

Levodopa induces sharp increases in striatal dopamine levels, which are particularly elevated in Parkinson’s disease patients who experience LIDs (Pagano et al. 2017a, b). However, a moderate-to-severe loss of dopaminergic terminals in the dorsal putamen is a necessary condition for the development of LIDs. This is associated with the inability of remaining dopaminergic terminals to remove the released dopamine and to store it into the pre-synaptic vesicles. In these circumstances, the same amount of levodopa administered induces higher release of dopamine in the extracellular space (augmented dopamine percent change from basal levels) (Lindgren et al. 2010). This results in higher swings in synaptic levels of dopamine and pulsatile stimulation of post-synaptic receptors located on striatal projection neurons (Pagano et al. 2017a, b). At the same time, in the absence of enough intact dopaminergic terminals, exogenous levodopa is metabolized in other terminals expressing the enzyme aromatic l-amino acid decarboxylase (AADC), such as serotonergic, which do not possess the molecular machinery to properly control the release of dopamine (Carta et al. 2010). Since serotonergic neurons lack of an auto-regulatory feedback of dopamine release, serotonergic terminals will release dopamine in a non-physiological manner. This results in higher swings in synaptic levels of dopamine and pulsatile stimulation of post-synaptic receptors located on striatal projection neurons (Carta and Bezard 2011).

This theory has been tested in preclinical models of Parkinson’s disease. The number of striatal serotonin terminals has been associated with both striatal dopamine peaks and abnormal involuntary movements (AIMs) scores in preclinical models of Parkinson’s disease induced by 6-hydroxy (6-OH) dopamine (Gil et al. 2011). In addition, it has been demonstrated that dopamine release from serotonergic terminals is ectopic in terms of both subcellular release sites and anatomical distribution. Rats lesioned by 6-OH dopamine showed a large increase in dopamine levels after levodopa administration in several brain areas, including hippocampus and prefrontal cortex, richly innervated by serotonergic pathways. These increases were totally abolished by a complete lesion of serotonin neurons (Navailles et al. 2010). AIMs are alleviated by lesioning the serotonergic system with 5,7-dihydroxytryptamine (Carta et al. 2007) and by blocking serotonergic transmission with 5-HT1A and 5-HT1B agonists (Bezard et al. 2013; Eskow et al. 2007; Munoz et al. 2008, 2009). However, glutamate terminals also express 5-hydroxytryptamine 1A (5-HT1A) and 5-HT1B receptors and the anti-dyskinetic of effect 5-HT1A and 5-HT1B agonists might be in part related to the reduction of striatal glutamate activation (Mignon and Wolf 2005; Munoz et al. 2008; Dupre et al. 2011). To discriminate the contribution of these two mechanisms, the effects of 5-HT1A and 5-HT1B agonists have been investigated on AIMs induced by levodopa and apomorphine (Munoz et al. 2009). They found that low doses of 5-HT1A and 5-HT1B agonists were able to suppress the mild levodopa-induced AIMs, but they did not reduce the moderate–severe apomorphine-induced AIMs (Munoz et al. 2009). These findings confirm that the activation of pre-synaptic receptor accounts for the effect of combined low doses of 5-HT1A and 5-HT1B agonists on mild dyskinesias, whereas other mechanisms may explain the more severe apomorphine-induced AIMs. In dyskinetic 6-OH, dopamine-lesioned rats have also been demonstrated that systemic administration of levodopa-induced AIMs and increased striatal glutamate levels; the administration of 5-HT1A receptor agonist 8-OH-DPAT was able to reduce both AIMs and the enhanced striatal glutamate levels (Dupre et al. 2011). This confirms the glutamatergic component of the effect of 5-HT1A agonist on dyskinesias. These results have been confirmed in the 1-methyl-4-phenyl-1,2,3,6-tetrahydropyridine (MPTP) monkey models of Parkinson’s disease (Bezard et al. 2013; Rylander et al. 2010). MPTP monkeys show increased serotonergic terminals in the striatum (Rylander et al. 2010) and a positive anti-dyskinetic effect of 5-HT1A and 5-HT1B agonist anpirtoline (Bezard et al. 2013). This evidence further confirms an abnormal sprouting of serotonin terminals in Parkinson’s disease, suggesting a key role of serotonergic system in the development of LIDs in Parkinson’s disease.

## PET imaging of serotonergic terminals in LIDs: in vivo human evidence

We performed a series of PET studies that have translated into humans these experimental observations. In the first PET study (Politis et al. 2014), we compared Parkinson’s patients with LIDs to those with stable response to levodopa in terms of serotonergic terminals density and striatal dopamine release. We measured serotonergic terminals using [^11^C]DASB PET imaging and dopamine release using [^11^C]raclopride PET, a D2 receptor antagonist radioligand which competes with endogenous dopamine for D2 receptor binding. Changes in D2 receptor availability, as reduction of baseline [^11^C]raclopride levels after levodopa administration, allow an indirect measure of synaptic dopamine release. Parkinson’s patients with LIDs showed increased dopamine release after the administration of levodopa compared to those with stable response to levodopa, with a relative preservation of serotonergic terminals in the putamen (Politis et al. 2014). Oral administration prior to levodopa of the 5-HT1A receptor agonist buspirone, a pre-synaptic modulator of serotonergic system, reduced levodopa-evoked striatal synaptic dopamine release and attenuated LIDs (Politis et al. 2014). To note, among patients with LIDs, the anti-dyskinetic effect of buspirone was greater in those with higher levels of serotonergic terminals, who also exhibited a greater decrease in dopamine release after buspirone pretreatment (Politis et al. 2014). We also divided the patients with LIDs into two groups based on the severity of LIDs severity (milder versus severe forms). We found that buspirone-associated modulation of dopamine levels was greater in patients with milder LIDs compared to those with more severe LIDs (Politis et al. 2014). This suggests that in Parkinson’s patients who experience more severe LIDs, higher doses or stronger 5-HT1A agonists are needed to achieve similar suppression of LIDs. Another possibility, however, is that other downstream mechanisms, such as glutamatergic overactivity, could play a more dominant role in generating severe dyskinesias, and therefore, the combined use of a 5-HT1A agonist and an NMDA antagonist may be needed. This is in line with the preclinical experimental evidence previously described (Bezard et al. 2013), in which 5-HT1A and 5-HT1B agonists were able to suppress the mild levodopa-induced AIMs, but not to reduce the moderate–severe apomorphine-induced AIMs (Bezard et al. 2013). Overall, the findings from this study provide the first human evidence that striatal serotonergic terminals contribute to LIDs pathophysiology via aberrant processing of exogenous levodopa and release of dopamine as false neuro-transmitter in the denervated striatum of Parkinson’s patients with LIDs.

We then investigated the role of serotonergic innervation of the globus pallidus in the development of dyskinesias (Smith et al. 2015). We measured the density of serotonergic terminals and the striatal dopamine release in the globus pallidus of Parkinson’s patients with LIDs compared to those with stable response to levodopa by using [^11^C]DASB PET and [^11^C]raclopride challenge, respectively. Parkinson’s patients with LIDs showed preserved serotonergic terminals in the globus pallidus, with a level similar to healthy controls. Higher density of serotonin terminals in the globus pallidus correlated with a greater amount of dopamine released and greater severity of LIDs. This indicates that either the serotonin terminal function in the globus pallidus in patients with LIDs is spared or that an adaptive terminal sprouting of remaining serotonergic projections occurs not only in the putamen but also in the globus pallidus. Taking together this finding (preserved serotonergic terminals in the globus pallidus) and the previous one (preserved serotonergic terminals in the putamen), we suggest that the imbalance caused by a normalization of serotonin terminals in the dopamine-denervated striatum creates increased dopamine release after levodopa administration, resulting in an increased negative input to the globus pallidus neurons controlling thalamic output. Greater LIDs might be the results of increased dopamine release at pre-synaptic dopaminergic receptors located at the synapses of striato-pallidal GABAergic neurons in the globus pallidus. These neurons control the projection neurons to the thalamus and thereby the thalamic output. By over-inhibition of these neurons, the dysregulated basal ganglia output then results in LIDs. This is in line with the preclinical evidence of a profound suppression of globus pallidus output activity in monkeys experiencing LIDs (Papa et al. 1999). However, it is uncertain how this would be different depending upon whether it is globus pallidus internal or globus pallidus external that is affected and whether pre-synaptic dopaminergic receptors would convey these effects. Further studies are needed to clarify this issue.

In the third study (Roussakis et al. 2016), the authors investigated the interaction between serotonergic and dopaminergic terminals in the development of LIDs. They measured the density of serotonergic and dopaminergic terminals in the striatum of Parkinson’s patients with LIDs and of patients with stable response to levodopa using [^11^C]DASB PET and [^123^I]FP-CIT SPECT, respectively. They found that higher putaminal serotonergic-to-dopaminergic terminals’ ratio correlates with longer disease duration in Parkinson’s patients, indicating that, as Parkinson’s progresses, the ratio between serotonergic and dopaminergic terminals becomes higher, as reflected by the higher [^11^C]DASB PET to [^123^I]FP-CIT SPECT binding ratio. This might be due to a faster disappearance of dopaminergic terminals compared to serotonergic ones, or to an aberrant sprouting of serotonergic innervation in the patients who will experience LIDs, as previously demonstrated in animal studies (Carta et al. 2007, 2010). In parallel, a fourth PET study from another team has also shown that, compared to non-dyskinetic patients, Parkinson’s patients with LIDs had a higher striatal serotoninergic-to-dopaminergic terminals availability, as reflected by the higher [^11^C]DASB to [^18^F]FP-CIT PET-binding ratio, with no difference in striatal dopaminergic terminals (Lee et al. 2015).

**Fig. 1:**
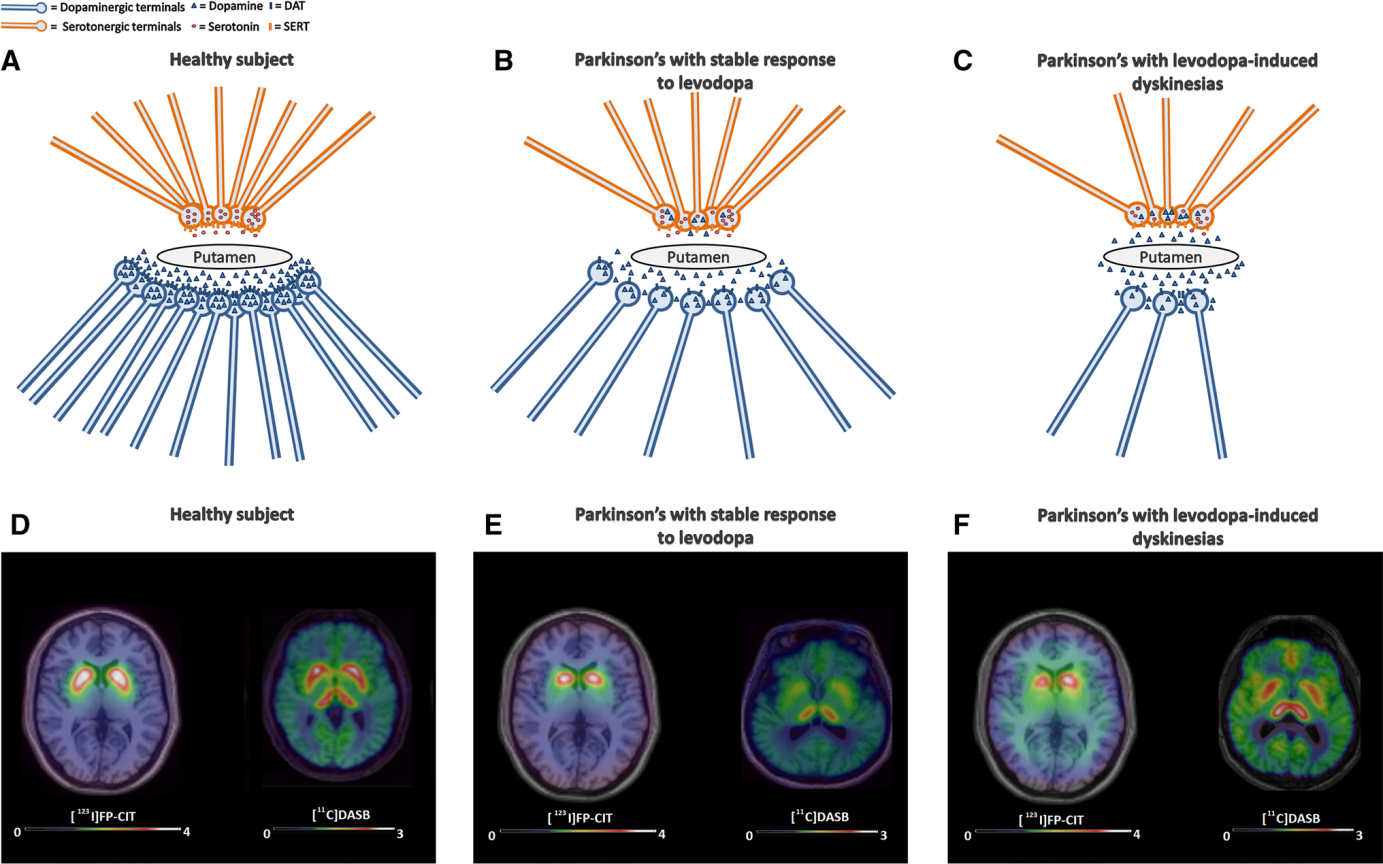
Involvement of the serotonergic system in the development of levodopa-induced dyskinesia. Illustration of the striatal serotonergic and dopaminergic innervation in healthy subjects (**a**), Parkinson’s patients with stable response to levodopa (**b**), and Parkinson’s patients who experiences levodopa-induced dyskinesias (**c**). **a** Healthy subjects showed normal dopaminergic and serotonergic terminals. **b** Parkinson’s patients with stable response to levodopa show loss of dopaminergic and serotonergic terminals. The remaining dopaminergic terminals reuptake dopamine through DAT and store it at pre-synaptic level. Serotonergic terminals do not release dopamine excessively after levodopa supplementation. As a consequence, dopamine levels remain relatively stable in the synaptic cleft and patients have a stable response to levodopa. **c** Parkinson’s patients who experience levodopa-induced dyskinesias show dramatic loss of dopaminergic terminals; however, the loss of serotonin terminals has not achieved such a critical low. The few remaining dopaminergic terminals lack the capacity to store dopamine and fail to reuptake dopamine through DAT. The serotonergic terminals try to compensate to the loss of dopaminergic terminals by handling the uptake and release of dopamine in the synapse. However, serotonergic terminals release dopamine in an excessive and inappropriate manner after levodopa supplementation, due to a lack of an auto-regulatory feedback for dopamine release in serotonergic neurons. As a consequence, there is a sharp and dysregulated increase of dopamine levels in the synaptic cleft, which is associated with the development of levodopa-induced dyskinesias. **c** Summed [^123^I]FP-CIT SPECT and [^11^C]DASB PET images coregistered and fused with 3T MRI images at the level of the dorsal basal ganglia for a healthy subject with normal dopaminergic and serotonergic innervation (**d**), a Parkinson’s patient with stable response to levodopa with loss of serotonergic and dopaminergic innervation (**e**), and a Parkinson’s patient who experiences levodopa-induced dyskinesia with a loss of dopaminergic innervation but preserved serotonergic innervation (**f**). *DAT* dopamine transporter, *SERT* serotonin transporter, *FP-CIT* SPECT ligand with high selectivity for DAT, *DASB* PET ligand with high selectivity for SERT

Overall, these findings suggest that when the dopaminergic innervation in the striatum and in the pallidum is critically low, the serotonergic system plays an important role in the development of LIDs. We have illustrated this in Fig. 1 with a cartoon showing the striatal serotonergic and dopaminergic innervation in healthy subjects (Fig. 1a), Parkinson’s patients with stable response to levodopa (Fig. 1b) and Parkinson’s patients who experiences levodopa-induced dyskinesias (Fig. 1c). Healthy subjects showed normal dopaminergic and serotonergic terminals, where Parkinson’s patients with stable response (Fig. 1b) to levodopa show loss of dopaminergic and serotonergic terminals. The remaining dopaminergic terminals reuptake dopamine through DAT and store it at pre-synaptic level. Serotonergic terminals do not release dopamine excessively after levodopa supplementation. As a consequence, dopamine levels remain relatively stable in the synaptic cleft and patients have a stable response to levodopa. Parkinson’s patients who experience levodopa-induced dyskinesias (Fig. 1c) show dramatic loss of dopaminergic terminals; however, the loss of serotonin terminals has not achieved such a critical low. The few remaining dopaminergic terminals lack the capacity to store dopamine and fail to reuptake dopamine through DAT. The serotonergic terminals try to compensate to the loss of dopaminergic terminals by handling the uptake and release of dopamine in the synapse. However, serotonergic terminals release dopamine in an excessive and inappropriate manner after levodopa supplementation, due to a lack of an auto-regulatory feedback for dopamine release in serotonergic neurons. As a consequence, there is a sharp and dysregulated increase of dopamine levels in the synaptic cleft, which is associated to the development of levodopa-induced dyskinesias. We also showed, in Fig. 1, in vivo measured of dopaminergic terminals, using [^123^I]FP-CIT SPECT, and serotonergic terminals, using [^11^C]DASB PET images coregistered and fused with 3T MRI images at the level of the dorsal basal ganglia for a healthy subject with normal dopaminergic and serotonergic innervation (Fig. 1d), a Parkinson’s patient with stable response to levodopa with loss of serotonergic and dopaminergic innervation (Fig. 1e), and a Parkinson’s patient who experiences levodopa-induced dyskinesia with a loss of dopaminergic innervation but preserved serotonergic innervation (Fig. 1f). These findings support the role of serotonergic terminals in the aberrant release of pallidal–striatal dopamine and in promoting the development of LIDs in patients with Parkinson’s disease.

## PET imaging of serotonergic terminals in GIDs: in vivo human evidence

Transplantation with foetal ventral mesencephalic tissue aims to restore the dopaminergic terminals in advanced cases of Parkinson’s disease. This treatment showed robust efficacy in some patients with remarkable improvement of motor symptoms but was also associated to severe adverse reactions consisting of developing troublesome involuntary movements when ‘off’ their dopaminergic drugs, called GIDs (Freed et al. 1990, 1992; Hagell et al. 2002; Levivier et al. 1997; Lindvall et al. 1990, 1992; Ma et al. 2002; Olanow et al. 2009; Peschanski et al. 1994; Widner et al. 1992). Graft tissue contained a varied proportion of non-dopaminergic cells including serotonergic neurons. Thus, striatal graft tissue containing high levels of serotonin neurons will lead to mishandling of striatal dopamine levels resulting in the occurrence of GIDs (Politis 2010; Politis et al. 2010, 2011). We have demonstrated that the same serotonergic mechanisms, such as excessive striatal serotonergic innervation and high serotonin-to-dopamine striatal terminal ratio, are pivotal in the development of GIDs in Parkinson’s patients who underwent striatal transplantation with foetal ventral mesencephalic tissue (Politis 2010; Politis et al. 2010, 2011).

In three Parkinson’s patients with GIDs who received striatal transplantation with foetal ventral mesencephalic tissue, we evaluated the density of serotonergic terminals, using [^11^C]DASB PET imaging, and the pre-synaptic ADCC dopaminergic activity, using [^18^F]dopa PET imaging. All three patients showed an excessive graft-derived serotonergic innervation (Politis et al. 2010) and high serotonin-to-dopamine terminal ratio (Politis et al. 2011). Furthermore, administration of small, repeated doses of 5-HT1A receptor agonist buspirone was able to attenuate graft-induced dyskinesias possibly by attenuating the abnormal serotonin terminal-derived dopamine release. These findings support the involvement of the serotonergic system in the development of GIDs and indicate that a ‘close-to-normal’ striatal serotonin/dopamine ratio in the transplanted foetal ventral mesencephalic tissue should be necessary to avoid the development of GIDs. However, it is important to underline that [^18^F]dopa PET binds ADCC also in serotonergic terminals and the ratio SERT/DAT should be better calculated using DAT-specific tracers (as we did in patients with LIDs).

Taking these results into account, we suggest that SERT/DAT ratios could be used as biomarker for LIDs and GIDs. Moreover, pharmacological modulation of serotonergic terminals sprouting, such as with brain growth factors, might also improve the severity of LIDs.

## Conclusions

In vivo PET studies confirmed in humans that an abnormal spreading of serotonergic terminals within the putamen has a pivotal role in the development of LIDs and GIDs. Serotonergic terminals’ release of abnormal amounts of dopamine after levodopa administration is the most widely recognized factor underlying the pathophysiology of dyskinesias but cannot be considered a necessary and sufficient condition for the development of this motor complication. The implementation of novel PET ligands is warranted for unveiling unexplored mechanisms of underlying pathophysiology of Parkinson’s disease. An integrative multimodal imaging approach combining different techniques is needed to untwist the debate around putative players on the pre-synaptic side of the cascade that lead to the development of dyskinesias in Parkinson’s disease.
